# Dynamics of Mpox infection in Nigeria: a systematic review and meta-analysis

**DOI:** 10.1038/s41598-024-58147-y

**Published:** 2024-03-28

**Authors:** Simeon Cadmus, Victor Akinseye, Matthias Besong, Tobi Olanipekun, John Fadele, Eniola Cadmus, Rashid Ansumana, Daniel Oluwayelu, Solomon O. Odemuyiwa, Oyewale Tomori

**Affiliations:** 1 Department of Veterinary Public Health and Preventive Medicine, University of Ibadan, Ibadan, Nigeria; 2 Damien Foundation Genomics and Mycobacteria Research and Training Centre, University of Ibadan, Ibadan, Nigeria; 3 Centre for Control and Prevention of Zoonoses, University of Ibadan, Ibadan, Nigeria; 4 Department of Chemical Sciences, Augustine University, Ilara-Epe, Lagos, Nigeria; 5 Federal Ministry of Agriculture and Food Security, Abuja, Nigeria; 6 Department of Community Medicine, College of Medicine, University of Ibadan, Ibadan, Nigeria; 7 School of Community Health Sciences, Njala University, Bo, Sierra Leone; 8 Department of Microbiology, University of Ibadan, Ibadan, Nigeria; 9 Department of Veterinary Pathobiology, University of Missouri, Columbia, MO USA; 10 African Centre of Excellence for Genomics of Infectious Diseases, Redeemer’s University, Ede, Osun State Nigeria; 11 Nigeria Institute of Medical Research, Yaba, Lagos, Nigeria

**Keywords:** Diseases, Medical research, Risk factors

## Abstract

The seasonal outbreaks of Mpox continue in most parts of West and Central Africa. In the past year, Nigeria had the highest number of reported cases. Here, we used the PRISMA guidelines to carry out a systematic review and meta-analysis of available evidence on Mpox in Nigeria to assess the prevalence, transmission pattern, diagnostic approach, and other associated factors useful for mitigating the transmission of the disease. All relevant observational studies in PubMed/MEDLINE, Embase, AJOL, Web of Science, Scopus and Google Scholar on Mpox in Nigeria were assessed within the last fifty years (1972 to 2022). In all, 92 relevant articles were retrieved, out of which 23 were included in the final qualitative analysis. Notably, most of the cases of Mpox in Nigeria were from the southern part of the country. Our findings showed a progressive spread from the southern to the northern region of the country. We identified the following factors as important in the transmission of Mpox in Nigeria; poverty, lack of basic healthcare facilities, and risk of exposure through unsafe sexual practices. Our findings reiterate the need to strengthen and expand existing efforts as well as establish robust multi-sectoral collaboration to understand the dynamics of Mpox Nigeria.

## Introduction

Mpox is caused by the Mpox virus (MPXV), an enveloped double-stranded DNA virus in the genus *Orthopoxvirus* within the *Poxviridae* family of viruses^1–3^. Before 2022, Mpox was a viral zoonosis believed to be endemic to West and Central African countries^4,5^. Since then, an unprecedented increase in cases has been observed in West Africa, along with reports in many other countries worldwide^6–9^. In 2023, 87,858 cases and 143 deaths were reported from 111 countries between January 1 and May 30^32^. A majority (n = 59,413, 67.6%) of these cases were reported in the Americas, 25,902 (29.5%) in Europe, 1794 (2%) in Africa, 608 in the Western Mediterranean region and 90 in the Eastern Pacific Region^32^. Consequently, the WHO declared Mpox a public health problem of international concern (PHEIC)^30,31^.

There are two distinct genetic clades of MPXV^1^: the Congo Basin clade (now renamed as Clade I) and the West African clade (renamed as Clade II)^4,10,11^. The disease was first identified in cynomolgus monkeys in Denmark in 1958^12^, and thus the name monkeypox. The first human case was reported several years later, in 1970, in the Democratic Republic of Congo (DRC)^13^. Outbreaks were subsequently reported in Sierra Leone, Liberia, and Nigeria, predominantly among children who had not received the smallpox vaccine^13,14^. Since then, outbreaks have been sporadically reported in the DRC, Nigeria, Ghana, and Cameroon^14–16^. However, the two decades between 2000 and 2019 saw an approximately tenfold increase from 2000 cases in the year 2000 to 19,000 reported cases in these countries^14,17,18^. The factors responsible for the current globalisation of Mpox are unknown.

Although the virus was first identified in monkeys, nonhuman primates are thought to be infected like humans^1,3,6,19^. Several rodents, including rope squirrels, tree squirrels, Gambian pouched rats, and dormice, have been suggested as natural hosts of the virus^1,2,20,21^. The Mpox virus is transmitted from animals to humans, mainly through direct contact with blood, body fluids, or cutaneous or mucosal lesions of infected animals^22,23^. Human-to-human transmission has also been reported via direct contact with infected materials from skin lesions of infected persons, through respiratory droplets following prolonged face-to-face contact, and through recently contaminated objects such as cloths and beddings^5,8,22^. Vertical transmission through the placenta and during childbirth has also been reported^22,23^. Additionally, the epidemiological patterns of recent outbreaks in Europe suggested a sexual route of transmission^5,24^. Although the mechanism of sexual transmission has not been fully elucidated^8,24–26^, recent reports have established sexual transmission^25,27–32^. Over 80% of cases were in men who had sex with men^32^.

The clinical symptoms of Mpox infection include fever, headache, muscle pains, general body weakness and lymphadenopathy, which are the main features of the first phase of the disease. The second phase, which manifests with rashes, usually begins 1–3 days after the onset of fever. Mpox infections resolve independently within 2 to 4 weeks in healthy individuals. More severe forms of the disease occur among children and immunocompromised individuals^29^.

Nigeria has the highest reported cases (842) of Mpox in Africa as of 19 May 2023, followed by DRC (739), Ghana (27), Central African Republic (30), and Cameroon (29)^32^. Since the re-emergence of the disease in Nigeria between September 2017 and August 7, 2022, a total of 985 suspected Mpox cases have been reported, with 398 (40.4%) confirmed and 12 deaths (CFR = 3.0%)^33^. Approximately 66.1% of the cases were males. Thirty of the 36 states and the Federal Capital Territory (FCT) have reported at least one case (Table 1)^14,34,35^. Most of the high-burden states in Nigeria are located within the forest belt of the country^34^. This finding is consistent with the known nidation of Mpox transmission in the forested regions of West and Central Africa^3,23,36^. However, most cases reported in Nigeria were in urban centres and cities, probably a reflection of the locations of large hospitals and diagnostic facilities in urban areas rather than an indication of a rural–urban geographical divide in case distribution^14,37^.

**Table 1:** States with at least one reported case of Mpox in Nigeria.

S. no.	State	No of cases per state
1	Abia	7
2	Adamawa	13
3	Akwa Ibom	8
4	Anambra	9
5	Bauchi	1
6	Bayelsa	55
7	Benue	2
8	Borno	3
9	Cross River	17
10	Delta	41
11	Ebonyi	2
12	Edo	19
13	Ekiti	2
14	Enugu	4
15	FCT	13
16	Gombe	3
17	Imo	15
18	Kano	5
19	Kastina	2
20	Kogi	2
21	Kwara	5
22	Lagos	50
23	Nasarawa	11
24	Niger	2
25	Ogun	3
26	Ondo	16
27	Oyo	9
28	Plateau	9
29	River	65
30	Taraba	5

It is widely believed that the number of Mpox cases is grossly underestimated since reports of cases which are asymptomatic have recently emerged^29^. In Nigeria, underreporting may result from factors such as a poor index of suspicion due to lack of awareness of healthcare workers, poor surveillance and weak health systems. Other factors include inadequate number of laboratories with the capacity to diagnose the disease, lack of access to approved antiviral medicines and vaccines, and stigmatisation^14,38–41^. Nigeria with a population of a median age of 18.6 years, most of whom have never been vaccinated against smallpox, has among the largest pools of individuals susceptible to Mpox in the world^42^. A large proportion of these vulnerable individuals are women and children^43^. The country also ranks among the highest in terms of the burden of HIV/AIDS, tuberculosis, hunger and malnutrition/undernutrition, malaria and insecurity^44–47^. These are all known risk factors for Mpox and many other infectious diseases^3,29,48,49^. Thus, a national strategy to interrupt local transmission of the disease is urgently needed. So far, the spread of the disease from Nigeria to Israel (2018), the United Kingdom (September 2018, December 2019, May 2021 and May 2022), Singapore (May 2019) and the United States (July and November 2021) have been documented^6,29,50–52^. Thus, continued local transmission of Mpox in Nigeria may have implications for global health. This study, therefore, sets out to assess the prevalence and distribution of Mpox cases, transmission patterns, impact of comorbidities and existing diagnostic protocols for Mpox in Nigeria. This is aimed at providing useful baseline information for effective policy development geared toward mitigating the spread of the disease in the country.

## Results

### Characteristics of studies

**Figure 1:**
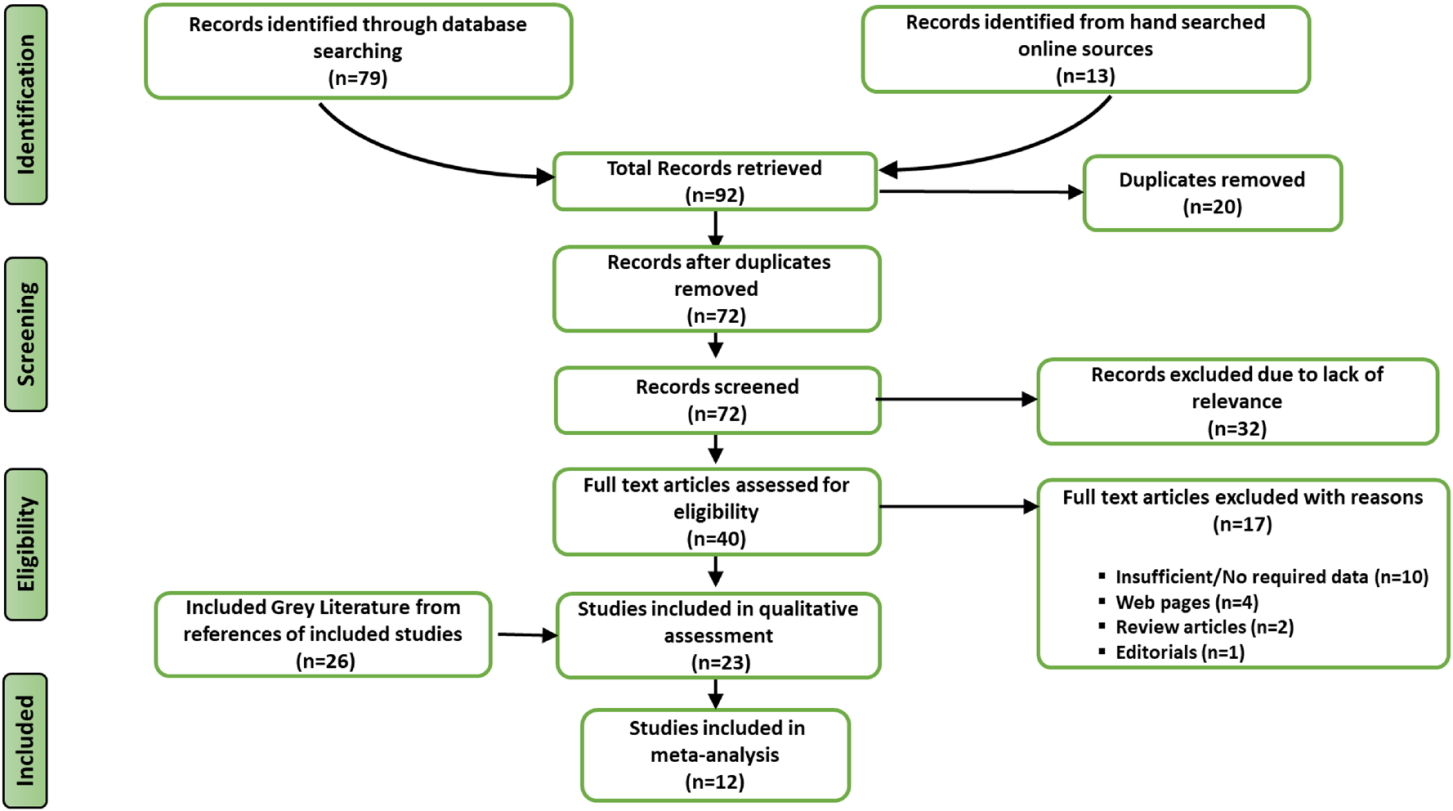
PRISMA flow chart showing the study selection process, Mpox systematic review, Nigeria, 2022.

A total of 92 articles were retrieved by literature search (79 from a database search and 13 from other sources). Of these, 72 articles were retained after duplicates were removed. Further, 32 were excluded due to irrelevance to the subject matter. The remaining 40 articles and abstracts were further screened based on the inclusion and exclusion criteria, and 17 were excluded. Finally, after a detailed full-text evaluation, 23 articles published between 1972 and 2022 were selected for qualitative analysis using the EPHPP. Only 12 of these articles were selected for quantitative analysis (Fig. 1). Of the 23 studies selected, 14 (60.9%) were case reports, six (26.2%) cross-sectional studies, one (4.3%) purposive cohort study, one case–control study (4.3%), and one (4.3%) retrospective cohort study. Eight (34.8%) of the selected studies were based on Mpox cases exported from Nigeria to other parts of the world. Fourteen (60.9%) of the cases were based solely on reports from the southern part of Nigeria, two (8.7%) were cases from northern Nigeria, and seven (30.4%) were based on cases reported across both the northern and southern parts of the country (Table 2). In addition to the included studies, information from 26 grey literature, obtained from the references of the included studies, were utilized in the review.

**Table 2:** Risk of bias assessment for the Mpox systematic review.

S. no	First author and year of publication	Population under consideration	Sample size adequacy	Subject and setting	Diagnostic tool deployed	Co-author accounted for	Subgroups identified	Quality items met
1	Yinka-Ogunleye, 2019	Yes	Yes	Yes	Yes	Yes	Yes	6
2	Ogoina, 2020	Yes	Yes	Yes	No	Yes	Yes	5
3	Erez, 2019	Yes	No	No	yes	Yes	No	3
4	Mauldin, 2022	Yes	Yes	Yes	Yes	Yes	Yes	6
5	Yong, 2020	Yes	No	No	Yes	Yes	No	3
6	Durski, 2018	Yes	Yes	Yes	No	Yes	No	4
7	Rao, 2022	Yes	No	No	Yes	Yes	No	3
8	Hobson, 2021	Yes	No	No	Yes	Yes	No	3
9	Costello, 2022	Yes	No	No	Yes	Yes	No	3
10	Vaughan, 2018	Yes	No	No	Yes	Yes	No	3
11	Amao, 2022	Yes	Yes	Yes	No	Yes	No	4
12	Ogoina, 2019	Yes	Yes	Yes	Yes	Yes	No	5
13	Eseigbe, 2021	Yes	No	Yes	Yes	Yes	No	4
14	Foster, 1972	Yes	No	Yes	Yes	Yes	No	4
15	Ogoina, 2022	Yes	No	Yes	Yes	Yes	No	4
16	Eteng, 2018	Yes	No	Yes	Yes	Yes	No	4
17	Pembi, 2022	Yes	No	Yes	Yes	Yes	No	4
18	Ogoina, 2022	Yes	No	Yes	Yes	Yes	No	4
19	Atkinson, 2022	Yes	No	No	Yes	Yes	No	3
20	Echekwube, 2020	Yes	Yes	Yes	Yes	Yes	Yes	6
21	Ita Ita, 2019	Yes	Yes	Yes	No	Yes	Yes	5
22	Chieloka, 2019	Yes	Yes	Yes	No	Yes	No	4
23	Ibegu, 2020	Yes	Yes	Yes	Yes	Yes	Yes	6

Across the six quality domains evaluated, all of the studies met three or more quality criteria, and most of the studies (n = 16) met four to six of the quality criteria assessed. The most common quality criteria failed by the studies were sample size, identification of subgroups, subjects and settings. All publications, however, passed the population under consideration criterion.

**Figure 2:**
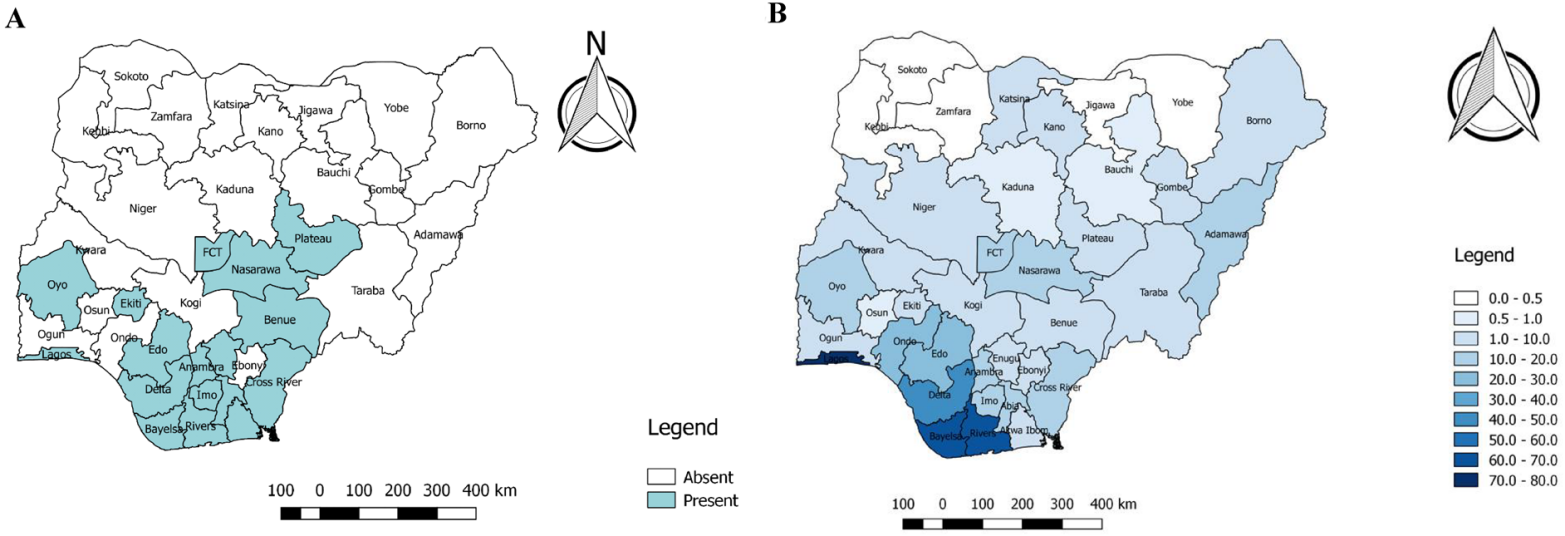
(**A**) Distribution of confirmed cases based on states between 2017 and 2018 in each geopolitical zone (N = 122). Data Source: NCDC, 2019. (**B**) Distribution of suspected Mpox cases in Nigeria between 2017 and 2022 (N = 503). Data Source: NCDC, 2022.

Between 2017 and 2018, 17 states in Nigeria reported at least one confirmed case of Mpox (Fig. 2A)^14^. All states in the country's South-south and Southeast geopolitical zone, except for Ebonyi State, reported at least one confirmed case of Mpox. Three states in the southwest (Lagos, Oyo, and Ekiti) and four in the North-central geopolitical zone (Plateau, Nasarawa, the Federal Capital Territory, and Benue) also reported at least one confirmed case. Nigeria's Northwest and Northeast geopolitical zones did not report any confirmed cases of Mpox between 2017 and 2018^14^.

According to the NCDC, as of August 28, 2022, thirty-two states in Nigeria had reported at least a confirmed case of Mpox in the country. All the states in the South-South, South-East, South-West, and North-Central geopolitical zones had recorded at least one case of Mpox^53^ (Fig. 2B). Also, five of the six states in the Northeast geopolitical zone have recorded a case of Mpox, with the exception of Yobe State. In contrast, only three (Kano, Katsina and Kaduna) of the seven states of the Northwest have recorded at least one case of Mpox. Zamfara, Kebbi, Jigawa, and Sokoto are the Northwest states yet to record a case of the disease^53^.

### Cases by gender, age, definition (suspected, confirmed) and outcome

Out of the 249 cases reviewed, 177 were males (71.1%) and 72 were females (28.9%)^14,54–68^. Those affected were between the ages of 1 year to 52 years, with the average age range between 4 and 40 years^14,54–57,60,62–65,69^. Eleven of the publications reviewed (47.8% [11/23]) recorded a mean age of 25 years and above, while two of the publications reported a mean age below 25 years (Table 3). Considering the number of suspected, confirmed, and fatal Mpox cases in selected peer-reviewed articles, it was observed that the entire 23 reviewed articles included information about suspected and confirmed cases as well as the number of confirmed cases in relation to the number of cases that resulted in the death of the patient involved (i.e., case fatality rate). Two hundred and twenty-six (48.2%) of the 469 suspected cases were confirmed to be Mpox, and only 13 (5.8%) of these 226 cases resulted in the death of the patients involved (Table 4).

**Table 3:** Studies included for analysis after full text evaluation, Mpox systematic review, Nigeria, 2022.

First author	Year of publication/study type	Diagnostic tool	Suspected cases	Confirmed cases	Fatality cases	Age range	Mean age	Male	Female	Location of detection	Mode of transmission	Co-infection
Adesola Yinka-Ogunleye	2019/Laboratory study	Molecular Method	276	122	7	0–50	29	84	38	Nigeria	NI	HIV
Ogoina D	2020/Retrospective	N.I.	51	40	5	0–52	32	31	9	Nigeria	NI	NI
Noam Erez	2019/Case investigation	Molecular Method	1	1	0	38	38	1	0	Israel	NI	NI
Matthew R. Mauldin	2022/Laboratory study	Molecular Method	5	5	0	32–40	37	4	1	U.K., Singapore, Israel	Rodent carcass, Occupational	NI
Sarah Ee Fang Yong	2020/Case investigation	Molecular Method	1	1	0	38	38	1	0	Singapore	Bushmeat	NI
Kara N. Durski	2018/Retrospective	N.I.	3	3	0	NI	NI	NI	NI	Nigeria	NI	NI
Agam K. Rao	2022/Case investigation	Molecular Method	1	1	0	NI	NI	1	0	USA	NI	NI
Gemma Hobson	2021/Case investigation	Molecular Method	1	1	0	NI	NI	1	0	UK	NI	NI
Varea Costello	2022/Case investigation	Molecular Method	1	1	0	28	28	1	0	USA	Direct Contact	N.I.
Aisling Vaughan	2018/Case investigation	Molecular Method	2	2	0	NI	NI	2	0	U.K	Consumption of bushmeat	N.I.
Lateefat Kikelomo Amao	2022/Laboratory study	N.I.	25	3	0	NI	NI	1	2	Nigeria	Direct Contact	NI
Dimie Ogoina	2019/Outbreak investigation	Molecular Method	38	18	0	NI	NI	17	1	Nigeria	N.I.	Syphilis, HIV, Chickenpox
E. E. Eseigbe	2021/Case investigation	Molecular Method	2	2	0	20	20	2	0	Nigeria	NI	NI
S. O. Foster	1972/Laboratory study	Microscopy	1	1	0	4	4	0	1	Nigeria	NI	NI
Dimie Ogoina	2022/Case investigation	Molecular Method	1	1	1	34	34	1	0	Nigeria	NI	NI
Womi-Eteng Eteng	2018/Outbreak investigation	Molecular Method, Serology	1	1	0	NI	NI	NI	NI	Nigeria	NI	NI
Emmanuel Pembi	2022/Case investigation	Molecular Method	1	1	0	30	30	1	0	Nigeria	NI	NI
Dimie Ogoina	2022/Case investigation	Molecular Method, Serology	1	1	0	NI	NI	NI	NI	Nigeria	NI	NI
Barry Atkinson	2022/Case investigation	Molecular Method, Serology	1	1	0	40	40	NI	NI	UK	NI	NI
Echekwube	2020/Case investigation	Molecular method	4	4	0	20–32	28		3	Benue, Nigeria	NI	HIV
Okonkon Ita Ita	2019/Laboratory study	N.I.	15	6	0	0–59	N.I.	8	7	Akwa Ibom, Nigeria	Bush meat, Rodent, Direct contact, sex	NI
Solomon Chieloka	2019/Outbreak investigation	N.I.	8	0	0	N.I.	25	NI	NI	Akwa Ibom, Nigeria	Bush meat consumption	NI
Ibegu	2020/Laboratory study	Molecular method	30	11	0	1–43	31	20	10	NI	NI	NI

*NI* not indicated.

**Table 4:** Mpox cases reported by gender, age and case definitions and outcome in publications under review.

S. no.	Author and year of publication	Gender		Age		Case definition		Outcome
		Male	Female	Age range	Average (in years)	Suspected	Confirmed	
1	Foster et al., 1972^76^	0	1	4	4	1	1	0
2	Ogoina et al., 2022^87^	16	5	22–43		NA	NA	NA
3	Durski et al., 2018^5^	NA	NA	NA	NA	3	3	0
4	Eteng et al., 2018^75^	NA	NA	NA	NA	1	1	0
5	Vaughan et al., 2018^61^	2	0	NA	NA	2	2	0
6	Erez, et al., 2019^55^	1	0	38	38	1	1	0
7	Ita Ita et al., 2019^67^	8	7	NA	NA	15	6	0
8	Ogoina et al., 2019^73^	17	1	NA	NA	38	18	0
9	Chieloka et al., 2019^70^	NA	NA	NA	25	8	0	0
10	Yinka-Ogunleye et al., 2019^14^	84	38	1 – 50	29	276	122	7
11	Echekwube et al., 2020^66^	1	3	20–32	28	4	4	0
12	Ibegu et al., 2020^68^	20	10	NA	NA	30	11	0
13	Ogoina et al., 2020^54^	31	9	1–52	32	51	40	5
14	Yong et al., 2020^57^	1	0	38	38	NA	NA	NA
15	Eseigbe et al., 2021^62^	2	0	20	20	2	2	0
16	Hobson et al., 2021^59^	1	0	NA	NA	1	1	0
17	Atkinson et al., 2022^69^	NA	NA	40	40	1	1	0
18	Amao et al., 2022^71^	1	2	NA	NA	25	3	0
19	Costello et al., 2022^60^	1	0	28	28	1	1	0
20	Mauldin et al., 2022^56^	4	1	32 – 40	37	5	5	0
21	Ogoin et al., 2022^64^	1	0	34	34	2	2	1
22	Pembi et al., 2022^65^	1	0	30	30	1	1	0
23	Rao et al., 2022^58^	1	0	NA	NA	1	1	0
	Total	177	72	NA	389	469	226	13

**Figure 3:**
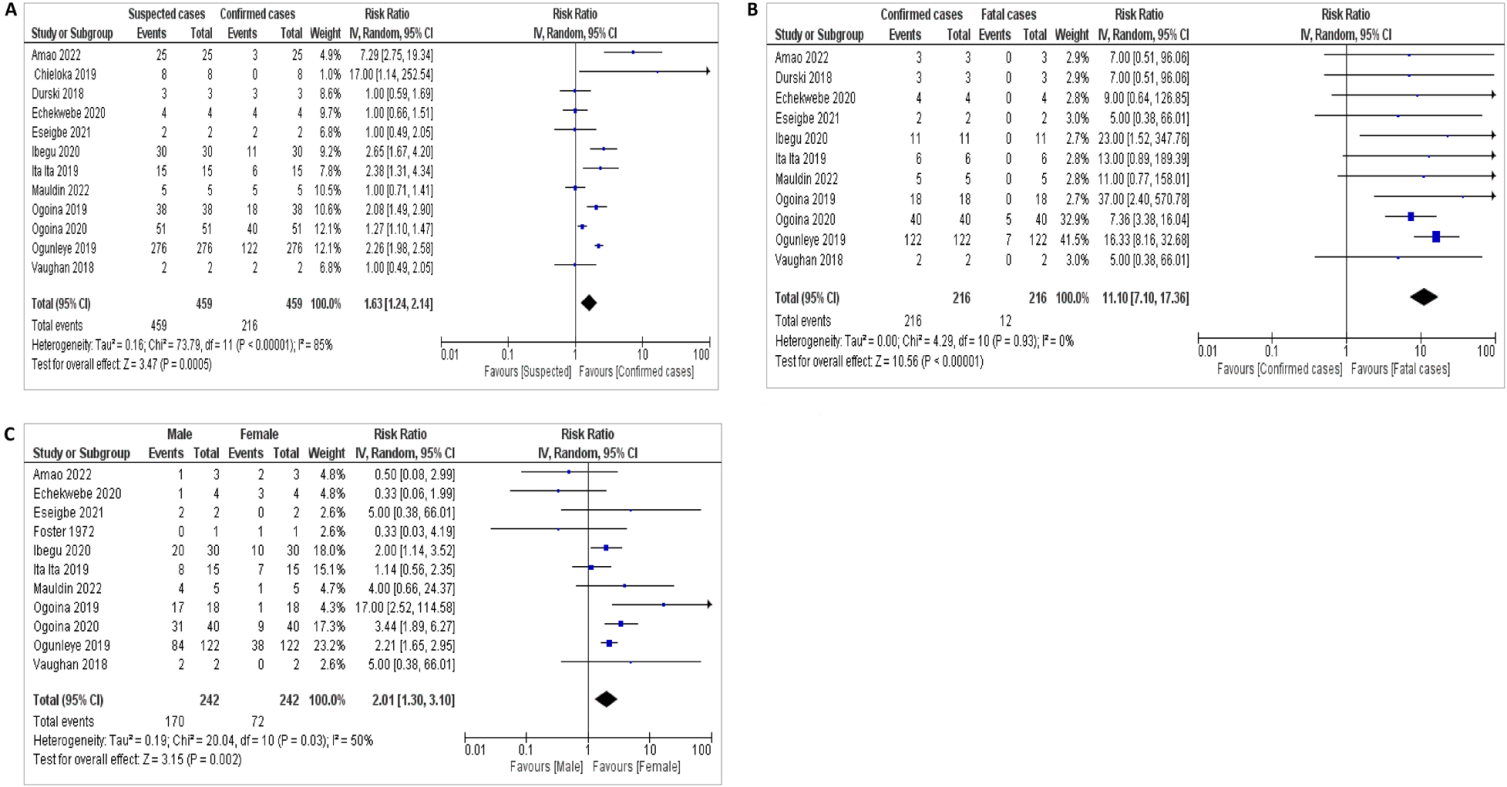
(**A**) Forest plot of the meta-analysis of suspected and confirmed cases. (**B**) Forest plot of the meta-analysis of confirmed cases and case fatality. (**C**) Forest plot of the meta-analysis of cases based on gender.

The studies considered for suspected and confirmed cases in Fig. 3A showed a high degree of heterogeneity, I^2^ = 85%. There exists a significant difference between the number of confirmed cases and suspected cases, and this was not due to chance, as shown by a statistical significance pooled estimate with a point estimate of 1.63 (CI: 1.24–2.14). This finding indicates that the majority of the suspected cases were not confirmed either due to diagnostic tools inadequacy or non-specificity of clinical signs (Fig. 3A).

The confirmed cases compared to the number of deaths in all the reviewed articles showed homogeneity, I^2^ = 0% (Fig. 3B). This finding shows that across all the studies considered, the rate of fatality reported compared to the confirmed cases is consistent regardless of the location. Further, the forest plot that compared the cases of Mpox between males and females showed a significant difference in the number of reported cases between the genders, and this was not due to chance. The reviewed studies also showed a calculated heterogeneity value (I^2^) of 50%, indicating average differences between studies. This is evident in the statistical significance of the pooled estimate with the point estimate (2.01) confidence interval (1.30–3.10) and a probability value (p = 0.002). This finding indicates a significant difference in cases reported between the two genders, with more cases reported in males (Fig. 3C).

### Poverty

Four reported cases of human infection with Mpox linked to individuals most of whom reside in the urban slums/sub-urban and rural areas were identified^62,66,70,76^. These included two female traders who reside in a rural area of southern Nigeria and presented to healthcare facility with numerous cutaneous eruption (papules, pustules and nodules)^66^. The others involved two cases of 20 a year old man and his step brother, both living in an urban slum in Northcentral Nigeria^62^. The first case, presented with a week’s history of fever, headache, pain on swallowing and micturition, and generalised skin lesions. The second, his step brother, and the primary care provider to the first case when he took ill, also presented with 1-week’s history of fever, headache, pain on swallowing, and skin lesions which were less intense than in the first case^62^. Notably, six cases of Mpox infection were reported in the rural/urban slums of Liberia, Nigeria and Sierra Leone between 1970–1971^76^, two of which were from Nigeria.

### Lack of basic healthcare facilities

Five studies associated with cases of Mpox infection and its severity to inadequate healthcare facilities including diagnosis and patients management^14,59,67,70,71^ were reviewed. Enhanced surveillance and strengthening of diagnostic tools/facilities have helped to improve the detection of more cases in real-time, especially in hotspot locations^14,70,71^. With optimal diagnostic facility, a man (the index case) and his family, including his wife travelled from southwestern Nigeria to the UK. The man, his wife and a caregiver were confirmed with Mpox infection following a PCR testing in the UK. As a result of the availability of required healthcare facilities, an in-depth contact tracing and active surveillance ensured that no transmission outside the index family occurred^59^. In contrast to the first outbreak of Mpox reported in Akwa Ibom, south-south of Nigeria, due to inadequate logistics for sample collection, transportation and diagnosis, needed active case detection and surveillance mechanisms could not be carried out. Unfortunately, only few suspected cases were assessed with limited samples collected, and those collected could not be processed due to logistics and diagnostics challenges^67^.

### Risk of exposure through unsafe sexual practices

Three studies reported the detection of highest proportion of human Mpox infection among sexually active age groups^67,68,87^. In two studies, more than half (53%) and about half (47%) of the entire study population were reported to be infected with Mpox cases, respectively^67,68^. Specifically, a study made a case for the role of sexual contact in the transmission of Mpox among confirmed cases from Nigeria. In the study, following the survey of sexual history of participants, and other associated risk behaviours and practices, 81.2% had genital ulcers, 56.2% reported to engage in high-risk behaviours (like unprotected sex, multiple sexual partners, and transactional sex) and 50% had sexual intercourse within a month before their first symptoms^87^.

### Mode of transmission

**Figure 4:**
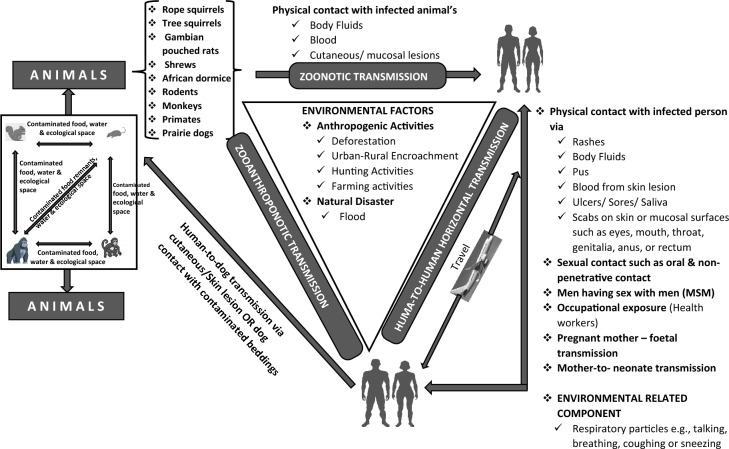
Overview of the transmission dynamics of Mpox virus infection.

Consumption of bush meat was the most prevalent mode of Mpox transmission recorded^56,57,61,67,70^. Other means of transmission included contact with rodent carcasses^56^, occupational risk (nosocomial)^56^, direct respiratory and mucosal contact with an infected person^60,67,71^, and sexual contact^57^. Other papers examined did not record a specific transmission mechanism^58,59^. Generally, various transmission routes have been reported, ranging from the most common animal-to-human transmission (zoonotic), human-to-human (horizontal transmission), human-to-animal (zooanthroponotic transmission), as well as animal-to-animal transmission^72^ (Fig. 4).

### Effect of concurrent infections/comorbidities

Four of the reviewed articles had information about co-infections. In the case of Mpox co-infection with HIV, a longer duration of disease, bigger lesions, severe cases of genital ulcers, and an increased risk of subsequent bacterial skin infection were observed^14,54,56,73^. In another instance in which a pregnant lady had Mpox, the infection led to premature membrane (amniotic sac) rupture and the subsequent vaginal delivery of a macerated fetus^73^.

### Diagnostic protocols utilised in the diagnosis of Mpox in articles under review

Exactly 13 (56.5%) of the reviewed articles reported the use of molecular techniques, namely the polymerase chain reaction (PCR)^14,55–60,62,64,65,69,73,74^ and seven (30.4) reported the use of DNA sequencing^14,56–59,61^ to identify, confirm and analyse the Mpox virus genome. Further, two studies employed the use of enzyme-linked immunosorbent assay (ELISA)^55,74^, and four other studies used IgM antibody testing^14,73–75^ for the detection of Mpox virus antibodies in clinical samples. The use of electron microscopy was reported in four studies^55,56, 69,76^, while the immunofluorescent assay^55^ and agar gel immunodiffusion test^76^ were the other diagnostic techniques reported in the articles reviewed.

### Exportation of Mpox from Nigeria to other parts of the world

Between 2018 and 2022, four countries reported the importation of Mpox cases from Nigeria. These are Singapore^56,57^, Israel^55,56^, the United Kingdom^56,59,61^, and the United States^58,60^.

## Discussion

A comprehensive study of the reviewed published literature on Mpox in Nigeria revealed several important facts. Nigeria occupies a central position in the current Mpox outbreak because researchers have traced the likely source of the outbreak to the country prior to 2017^14^. This single but important fact is a cause for concern considering the inconsistency and structural deficiency in the healthcare system of the country that has made the execution of preparedness plans, prompt response to disease outbreaks as well as robust contact tracing difficult to achieve.

It should be noted that Mpox infection is on the rise mostly in low- and middle-income countries of Africa due in part to the unavailability of vaccines, lack of information among at-risk people, or fear of seeking information due to the criminalisation of gay sex. Although researchers around the world have posited that Mpox may not cause any massive magnitude of infections, however, the disease has come to stay and eradicating it may be difficult due to constant mutations by the virus^77^. However, according to Tomori and Ogoina, the consequences of neglecting a disease anywhere around the world could be costly because a disease anywhere can spread everywhere if not given the required attention^78^.

This review revealed that despite inadequacies of the disease surveillance system in Nigeria, the cases reported have been on the increase since 2017. In addition, majority of the cases reported seem to be spillovers from animal hosts and not necessarily from outside the country. Thereafter, the infection has continued to spread within the human population. Previous studies have identified spillover hosts and poor disease surveillance as major culprits responsible for the continuous spread of the disease in Nigeria^79,80^.

The majority of Mpox cases in Nigeria were reported in the southern region of the country. This finding is consistent with the previous work that established the connection between Mpox transmission and the rainforest environment^79^. Notably, the availability of better disease surveillance and diagnostic facilities and infrastructure could be responsible for this finding. It is, however, important to note that before the 2005 outbreak in South Sudan, it was believed that Mpox was confined to the rainforest regions^81^. Importantly, the studies also revealed a progressive spread of the disease from the south to the northern part of Nigeria. In 2018, although most states in the southern region had already recorded at least a case of Mpox, only a few northern states had reported cases of the disease. Currently, as at the time of this review in 2023, the majority of states in northern Nigeria, especially in the north-central and northeastern regions, have also recorded at least one case of Mpox^52,82^, indicating a remarkable spread in the cases of Mpox within a short period.

The northern region of Nigeria is mostly semi-desert to desert (i.e., Guinea and Sudan savannah) in terms of vegetation, whereas the southern region is rainforest. Consequently, it is anticipated that the disease will spread from locations adjacent to the natural habitats of reservoir animals to regions that are far from such habitats. Therefore, it may be hypothesised that the majority of cases in the north are a result of direct or indirect contact with people who have contracted the disease and not due to direct contact with reservoir animals. Similarly, most cases were reported in the rural areas of the country^71^. This finding establishes a connection between poverty and lack of access to basic amenities in Nigeria and the spread of Mpox, as previously reported^83^.

Although the population of individuals affected by Mpox in Nigeria belong to a wide age range, most of the cases were reported among adults 25 years (about 85%) and above. The detection of cases among a few married sexual partners and the high rates of genital ulcers suggest a role of sexual contact in the transmission of MPX during the 2017 outbreak in Nigeria^14^. Further, most cases were reported among young to adult males. This finding indicates that the likelihood of males contracting Mpox in Nigeria is significantly greater than the likelihood of females contracting the disease.

The high incidence of Mpox among sexually active males in Nigeria can be partly explained by the recent surge in male-to-male sexual intimacy among Africans^84^ and is consistent with the reported incidence of Mpox among adult males with multiple sexual partners in developed nations^85^. Also, the likelihood of a man contracting the disease during a male-to-male sexual act is greater than the likelihood of disease transmission through a female-to-male sexual encounter, owing to the prolonged shedding of the virus in seminal fluid^7,86,87^. The issue of men who have sex with men (MSM) as a major risk factor for the increase in the spread of Mpox in Nigeria requires further investigation.

Socio-cultural practices play a crucial part in the transmission of Mpox. Many Nigerians consume bush meat, which is sometimes either improperly or unhygienically prepared. Since most of the animals that have been linked to Mpox transmission are associated with the wild, they could serve as reservoir/spillover hosts for the virus^88^. It is therefore not strange that higher incidence of the disease is being reported in the southern part of the country where wild animals are abundant due to the presence of forests, an ideal habitat for these animal reservoirs^82^. The exportation of the disease from Nigeria to other nations, such as the United Kingdom, Singapore, Israel, and the United States, as documented in this review, supports the transboundary nature of the Mpox virus^55–57^.

Confirmation of Mpox in Nigeria is based mainly on the use of molecular techniques such as PCR and genome sequencing. However, the equipment required for these procedures are not widely distributed throughout the country but are limited to a few referral laboratories in the urban centres. The use of these molecular methods provides a definitive diagnosis of the Mpox cases in Nigeria, although several cases might remain undiagnosed/undetected. Other diagnostic methods used include IgM antibody detection, electron microscopy, ELISA, immunofluorescent assay, and agar gel immunodiffusion test.

Overall, not all suspected cases of Mpox were confirmed to be the disease in Nigeria. This demonstrates the existence of other diseases with similar clinical manifestations and symptoms to Mpox in Nigeria. It is, therefore, essential to pay close attention to the occurrence of diseases with symptoms similar to those of Mpox among Nigerians. Some of these diseases include measles, chickenpox, smallpox, and other skin infections that present with skin rashes and related lesions.

Compared to the number of confirmed Mpox cases, the case fatality rate reported in the reviewed articles was low (5.8%). This finding further demonstrates that Mpox is a self-limiting disease, with only immunocompromised individuals experiencing the severe form^89^. The worsening of Mpox symptoms in the presence of co-infection with other immunocompromising diseases like HIV, TB and hepatitis supports the notion that, although the disease is self-limiting, a healthy immune system is essential to limit its progression and halt it within the expected two to four weeks^90^.

The findings of this review further give credence to the fact that immunocompromised individuals are more likely to suffer from the severe form of Mpox. Further, it was observed that contracting Mpox infection during pregnancy can cause the early rupture of the amniotic sac and the subsequent delivery of a mummified fetus. This assertion was further substantiated by studies that revealed the teratogenic effect of pox viruses^91,92^.

## Conclusion

There is a huge gap in knowledge on the current Mpox outbreak and control situation in Nigeria. Nigeria plays a key role in the exportation of Mpox to other countries. Hence, understanding the dynamics of the disease will help in solving other emerging/re-emerging infectious disease emergencies in Nigeria and in other countries with similar socio-cultural and ecological settings, as well as prevent future international spread of these diseases. Efforts are ongoing to perform genomics sequencing of all Mpox-positive samples by the NCDC. However, more still needs to be done by the NCDC in the area of coordination of already existing disease diagnostic infrastructures by getting the states more involved in surveillance systems. This will provide information on the source of index, transmission pattern, major drivers of the infection (socioecological factors), and the spread of the infection/disease and supplement efforts by the NCDC. Furthermore, there is still a need for robust multidisciplinary/multi-sectoral collaboration between researchers within and outside the country to find answers to some pertinent questions relating to the animal reservoirs of Mpox. These initiatives will help provide insight into the mode of transmission, presentation (especially viz-a-viz the possibility of asymptomatic carriers) and the need for partnerships to build a robust community-driven surveillance network for early detection, early response and reporting of cases of Mpox and other epidemic-prone diseases.

## Methods

A systematic assessment of published studies and reports on human Mpox cases in Nigeria was carried out based on the PRISMA recommendation. We searched for publications in PubMed/MEDLINE, Embase, AJOL, Web of Science, Scopus and Google Scholar for studies (full articles and abstracts) published within 1972 to 2022, involving the prevalence or incidence of human Mpox in Nigeria (and those with links to Nigeria). Key search words were used without regard to language. We also searched the internet for the study headings, titles, or abstracts (Supplementary Information).

### Search strategy

The main search strategy used was: ((“Monkeypox epidemiology”[All Fields] OR ((“monkeypox”[MeSH Terms] OR “monkeypox”[All Fields]) AND (“epidemiology”[MeSH Subheading] OR “epidemiology”[All Fields] OR “surveillance”[All Fields] OR “epidemiology”[MeSH Terms] OR “surveillance”[All Fields] OR “surveillances”[All Fields] OR “surveilled”[All Fields] OR “surveillance”[All Fields]))) AND (“nigeria”[MeSH Terms] OR “nigeria”[All Fields] OR “nigeria s”[All Fields])) OR ((“monkeypox”[MeSH Terms] OR “monkeypox”[All Fields] OR “Monkeypox outbreak”[All Fields]) AND “nigeria*”[All Fields]).

### Grey literature

All relevant information from government and international organisations’ websites and repositories were obtained from the references of the reviewed articles that met our inclusion criteria, and were categorised as grey literature. These materials, though relevant, did not meet our inclusion criteria because they were not articles published in peer reviewed journals, rather they are periodic information in the website of these international/national organisations/bodies. In all 26 articles were obtained from grey literature comprising eight (8) from World Health Organization, WHO; nine (9) from Nigeria Centre for Disease Control, NCDC; and two (2) from the European Centre for Disease Prevention and Control, ECDC. Others included two (2) from Nigeria Scholars; one (1) each from the websites of Nigeria Population Commission, NPC; Nigeria HIV/AID Indicator & Impact Survey, NAIIS; UNICEF; CDC; and National Action Plan for Health Security, NAPHS. These grey literature were included based on the relevance of their information to the topic under review.

### Selection strategy

The initial selection by title and abstract was conducted independently by two researchers: MB and TO according to the inclusion/exclusion criteria. All articles that presented one or more terms with Mpox and Nigeria relationship were included. Subsequently, an exhaustive reading of the articles was carried out to confirm inclusion of relevant data for the systematic review and important variables for the meta-analysis. The final decision on articles to be included was discussed with a third investigator (VA), and a consensus was reached. The PRISMA model was used to organise the information from the article selection process.

### Inclusion and exclusion criteria

The final reviewed articles and abstracts included prospective observational studies, case reports (including exported cases, i.e. those that have links/sources linked with Nigeria), cohort studies, and epidemiological investigations that reported on Mpox in humans were included. Resources excluded include full books, book sections, studies describing study populations not based in Nigeria, studies on animals or insects, studies not focused on Mpox patients exclusively, retrospective studies, reviews, editorials and publications lacking original data (transitory website-based information). Likewise, conference abstracts, conference proceedings, and review articles were excluded from the analysis.

### Data extraction

Using the inclusion and exclusion criteria, three reviewers (MB, TO and JF) independently screened the titles and abstracts of eligible studies using the Rayyan screening tool^93^. The whole text of the citations chosen for evaluation was obtained, and the reviewers independently collected all study data and resolved disagreements by consensus. The extracted data from each article included the first author, year of publication, study location, study enrolment period, number of suspected cases and the number of confirmed cases. Other information collected included the technique of diagnosis, comorbidity and case fatality rate. Other data obtained included age and gender of study participants/case reported, study participants, sample size and reported incidence/prevalence with 95% confidence intervals (CIs). If available, data on diagnostic protocol, transmission pattern, and comorbidities were included. The three reviewers also performed data extraction and resolved all disagreements by consensus.

### Quality assessment and risk of bias

The quality assessment of the study was carried out using the Effective Public Health Practice Project (EPHPP), a quality assessment tool for quantitative studies^94^. This tool ensures the evaluation of the risk of bias rather than excluding low quality literature. The risk of bias in each retrieved document was evaluated based on the population considered, sample size adequacy, study settings, diagnostic tool used, and subgroup reported.

#### Selection bias

This refers to the individuals who participated in the study under consideration.

Strong/moderate—if the individuals are solely representative of the prevalence or incidence of human Mpox in Nigeria; and more than 60% of the selected individuals agreed to participate in the study.

Weak—if the study is based on individuals from studies of diseases other than Mpox and if neither the research participants nor the occurrence can be traced to Nigeria.

#### Subject and setting

This pertains to the type of study being evaluated by the article under review.

Strong/moderate—if the article is based on cohort studies, observational studies, epidemiological studies, and case reports conducted on Mpox in Nigeria.

Weak—if the article is a book, book section, or research describing non-Nigerian populations i.e. participants who were believed to have contracted Mpox in Nigeria before going to other nations.

#### Diagnostic tool deployed

This relates to the dependability of the diagnostic techniques deployed.

Strong/moderate—if molecular techniques were utilised in the diagnosis of Mpox and if more than one diagnostic technique was used.

Weak—if Mpox was diagnosed using non-molecular techniques.

#### Subgroups identified

Strong/moderate—provided the study acknowledged the presence of distinct subgroups, such as gender and age.

Weak—if the study does not identify subgroups.

The studies that were identified as been weak based on the above described criteria were considered to show high level of bias and those that fell within the strong/moderate category were adjudged low and moderately biased. These were the studies included in the qualitative and quantitative analysis.

### Operational definitions

#### Suspected cases

We identified suspected cases as those with sudden onset of high fever, followed by a vesicular-pustular eruption showing predominantly on the face, palms of the hands, and soles of the feet or the presence of at least five scabs resembling smallpox lesions.

#### Confirmed cases

We identified confirmed cases as suspected cases confirmed by laboratory tests or analysis (positive IgM antibody, PCR, or virus isolation).

#### Probable cases

A suspected case with an epidemiological connection to a confirmed case but no chance for laboratory confirmation.

#### Possible cases

A case with a vesicular, pustular, or crusted rash that was not identified as chickenpox by the patient's family or physician.

Fever with vesicular or crusty rash prior history.

Individuals with unexplained rash, fever, and at least two additional clinically relevant symptoms in addition to meeting one of the epidemiological criteria or exhibiting increased levels of orthopoxvirus-specific IgM.

### Statistical analysis

Data were analysed using Revman Review Manager Version 5.4 software. The calculated results were presented in tables and graphs using descriptive statistics. The heterogeneity across studies was evaluated by Cochrane’s *Q*-test and I^2^ statistics, using the forest plot. The calculated value of I^2^ allows measuring the percentage of variability due to heterogeneity rather than chance difference or sampling error. If the value of I^2^ was greater than 60% and the Q test yields P < 0.05, heterogeneity was considered statistically significant. The random effects model, based on the DerSimonian–Laird method, which calculates the variability within and between studies, was applied to estimate the pooled prevalence of the various variables (case type and gender) and 95% CIs.

### Supplementary Information

Supplementary Information.

## Data Availability

All the data for this study will be made available on request. For request of data from this study, please contact: Simeon Cadmus (simeonc5@gmail.com); or Victor Akinseye (akinseye.toyin@gmail.com).
