# Supplementary material for: Dynamics of Mpox infection in Nigeria: a systematic review and meta-analysis
Source: Sci Rep. 2024 Mar 28;14:7368. doi: 10.1038/s41598-024-58147-y (PMC10978922; doi:10.1038/s41598-024-58147-y)
Supplement: Supplementary file 1 — Supplementary Information. [file 41598_2024_58147_MOESM1_ESM.docx]

PRISMA Checklist

| **Section and Topic** | **Item #** | **Checklist item** |
| --- | --- | --- |
| **TITLE** | | |
| Title | 1 | **Dynamics of Mpox Infection In Nigeria: A Systematic Review and Meta-Analysis** |
| **ABSTRACT** | | |
| Abstract | 2 | **The seasonal outbreaks of Mpox continues in most parts of West and Central Africa. In the past year Nigeria had the highest number of reported cases. Here, we used the PRISMA guidelines to carry out a systematic review and meta-analysis of available evidence on Mpox in Nigeria. All relevant observational studies in PubMed/MEDLINE, Embase, AJOL, Web of Science, Scopus and Google Scholar on Mpox in Nigeria within the last five years were assessed. In all 92 relevant articles were retrieved, out of which 23 were included in the final qualitative analysis. Over the past five years, thirty-two out of 36 states in Nigeria, including the Federal Capital Territory have reported at least a case of Mpox. Most of the cases of Mpox in Nigeria were from the southern part of the country. Our findings showed progressive spread from southern to the northern regions of the country. Further, we identified a positive association between infection and poverty, lack of basic healthcare facilities as well as multiple heterosexual partners. Our findings reiterate the need to strengthen and expand on the already existing efforts as well as establish robust multi-sectoral collaboration to understand the dynamics of Mpox Nigeria.** |
| **INTRODUCTION** | | |
| Rationale | 3 | **The rationale for the review is that It is widely believed that the number of Mpox cases is grossly underestimated since reports of cases with uncharacteristic signs and symptoms have recently emerged. In Nigeria, underreporting may result from factors such as poor index of suspicion due to lack of awareness of healthcare workers, poor surveillance, weak health system, inadequate laboratories with capacity to diagnose the disease, lack of access to approved antiviral medicines and vaccines, and stigmatization. The country ranks among the highest in terms of the burden of HIV/AIDS, tuberculosis, hunger and malnutrition/undernutrition, burden of malaria and insecurity. These and many other known risk factors for Mpox and many other infectious diseases abound in Nigeria. Thus, a national strategy to interrupt local transmission of the disease is urgently needed.** |
| Objectives | 4 | **This study was carried to assess published literature on human Mpox cases in Nigeria in order to provide the global community with information on the dynamics and possible drivers of the disease transmission.** |
| **METHODS** | | |
| Eligibility criteria | 5 | **The final reviewed articles and abstracts included prospective observational studies, case reports (including exported cases), cohort studies, and epidemiological investigations that reported on Mpox in humans were included. Resources excluded include full books, book sections, and studies describing study populations not based in Nigeria, studies on animals or insects, studies not focused on Mpox patients exclusively, retrospective studies, reviews, editorials and publications lacking original data (transitory website-based information). Likewise, conference abstracts, conference proceedings, and review articles were excluded from the analysis.** |
| Information sources | 6 | **We searched for publications in PubMed/MEDLINE, Embase, AJOL, Web of Science, Scopus and Google Scholar for studies (full articles and abstracts) published within the last five years, January 2017 to 2022, involving the prevalence or incidence of human Mpox in Nigeria (and those with links to Nigeria). Key search words were used without regard to language. We also searched the internet for the study headings, titles, or abstracts.** |
| Search strategy | 7 | **The main search strategy used was: (("Monkeypox epidemiology"[All Fields] OR (("monkeypox"[MeSH Terms] OR "monkeypox"[All Fields]) AND ("epidemiology"[MeSH Subheading] OR "epidemiology"[All Fields] OR "surveillance"[All Fields] OR "epidemiology"[MeSH Terms] OR "surveillance"[All Fields] OR "surveillances"[All Fields] OR "surveilled"[All Fields] OR "surveillence"[All Fields]))) AND ("nigeria"[MeSH Terms] OR "nigeria"[All Fields] OR "nigeria s"[All Fields])) OR (("monkeypox"[MeSH Terms] OR "monkeypox"[All Fields] OR "Monkeypox outbreak"[All Fields]) AND "nigeria*"[All Fields]).** |
| Selection process | 8 | **The initial selection by title and abstract was conducted independently by two researchers MB and OA according to the inclusion/exclusion criteria. In selecting articles' titles, all articles that presented one or more terms with Mpox and Nigeria relationship were included. After this, an exhaustive reading of the articles was carried out to confirm the relevant data for the systematic review and important variables for the meta-analysis. The final decision on articles to be included was discussed with a third investigator (VA), and a consensus was reached.** |
| Data collection process | 9 | **Using the inclusion and exclusion criteria, two reviewers (MB and OO) independently screened the titles and abstracts of eligible studies using the Rayyan screening tool. The whole text of the citations chosen for evaluation were obtained, and the reviewers independently collected all study data and resolved disagreements by consensus.** |
| Data items | 10a | **The extracted data from each article included the first author, year of publication, study location, study enrolment period, number of suspected cases and the number of confirmed cases. Other information collected included the technique of diagnosis, comorbidity and case fatality rate. Other data obtained included age and gender of study participants/case reported, study participants, sample size and reported incidence/prevalence with 95% confidence intervals (CIs), if available.** |
|  | 10b |  |
| Study risk of bias assessment | 11 | **The quality assessment of the study was carried out using the Effective Public Health Practice Project (EPHPP), a quality assessment tool for quantitative studies [https://www.ephpp.ca/quality-assessment-tool-for-quantitative-studies/]. This tool ensures the evaluation of the risk of bias rather than excluding low quality literature. The risk of bias in each retrieved document was evaluated based on the population considered, sample size adequacy, study settings, diagnostic tool used, and subgroup reported.** |
| Effect measures | 12 | **Population under consideration, sample size, subject and setting, diagnostic tool deployed, co-authors accounted for, and sub-group identified.** |
| Synthesis methods | 13a | **Not Applicable** |
|  | 13b | **Data were analysed using Revman Review Manager Version 5.4 software.** |
|  | 13c | **The calculated results were presented in tables and graphs using descriptive statistics.** |
|  | 13d | **Studies with low risk of bias based on the quality assessment criteria developed were included in the meta-analysis. The study must document reports for patients demography, confirmed cases, suspected cases and number of death.** |
|  | 13e | **The heterogeneity across studies was evaluated by Cochrane's Q-test and I^2^ statistics. The calculated value of I^2^ allows measuring the percentage of variability due to heterogeneity rather than chance difference or sampling error. If the value of I^2^ was greater than 60% and the Q test yields P < 0.05, heterogeneity was considered statistically significant.** |
|  | 13f | **Not applicable** |
| Reporting bias assessment | 14 | **The risk of bias assessment was carried out using by comparing each eligible study with five developed criteria based on the information retrieved from the assessed literature. The criteria include population under consideration, sample size, subject and setting, diagnostic tool deployed, and sub-group identified. The determination of the risk of bias is as follows:**  **1. Population in consideration: This refers to the individuals who participated in the study under consideration.**  **Yes - if the individuals are solely representative of the prevalence or incidence of human Mpox in Nigeria.**  **No - if the study is based on individuals from studies of diseases other than Mpox and if neither the research participants nor the occurrence can be traced to Nigeria.**  **2. Sample size adequacy: Adequate sample size refers to the number of participants in the study under consideration.**  **Yes - if the sample size is sufficient to identify disparities and subgroups among the persons under examination.**  **No - if the sample size is insufficient to distinguish between participants.**  **3. Subject and setting: This pertains to the type of study being evaluated by the article under review.**  **Yes – if the article is based on cohort studies, observational studies, epidemiological studies, and case reports conducted on Mpox in Nigeria.**  **No – if the article is a book, book section, or research describing non-Nigerian populations i.e. participants who were believed to have contracted Mpox in Nigeria before going to other nations.**  **4. Diagnostic tool deployed: This relates to the dependability of the diagnostic techniques deployed.**  **Yes - if molecular techniques were utilised in the diagnosis of Mpox and if more than one diagnostic technique was used.**  **No - if Mpox was diagnosed using non-molecular techniques.**  **5. Subgroups identified**  **Yes - provided the study acknowledged the presence of distinct subgroups, such as gender and age.**  **No - if the study does not identify subgroups.** |
| Certainty assessment | 15 | **The quality assessment of the study was carried out using the Effective Public Health Practice Project (EPHPP), a quality assessment tool for quantitative studies** |
| **RESULTS** | | |
| Study selection | 16a | 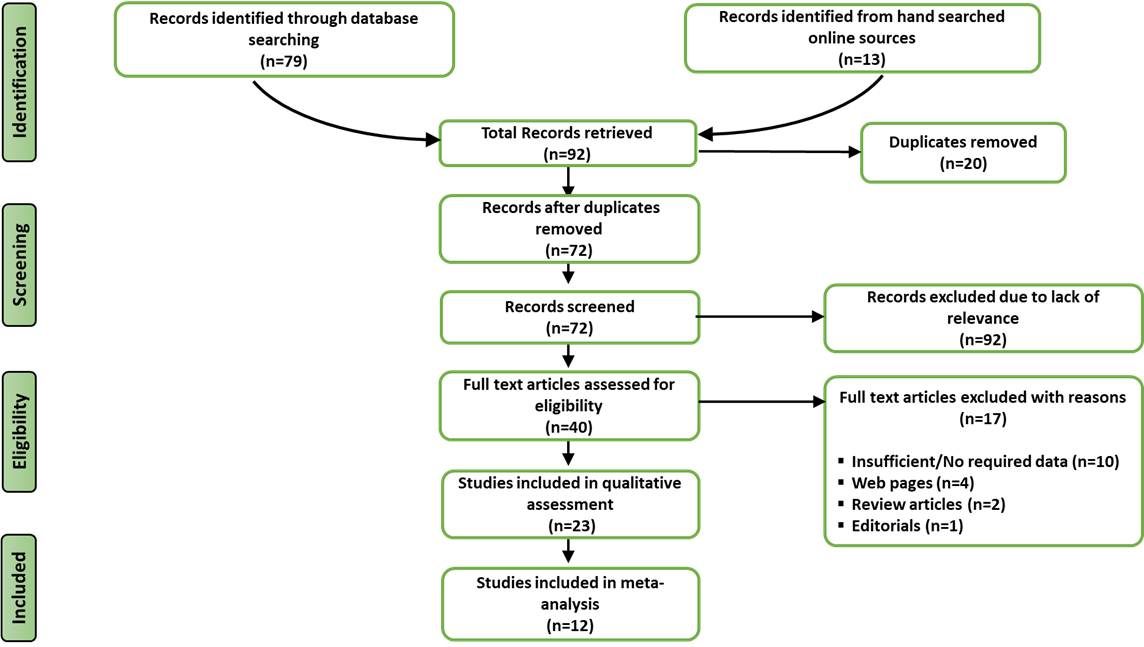 |
|  |  |  |
| Study characteristics | 17 | \| **First Author** \| **Year of Publication/ Study Type** \| **Diagnostic tool** \| **Suspected Cases** \| **Confirmed Cases** \| **Fatality cases** \| **Age range** \| **Mean Age** \| **Male** \| **Female** \| **Location of Detection** \| **Mode of Transmission** \| **Co-infection** \| \| --- \| --- \| --- \| --- \| --- \| --- \| --- \| --- \| --- \| --- \| --- \| --- \| --- \| \| Adesola Yinka-Ogunleye \| 2019/ Laboratory study \| Molecular Method \| 276 \| 122 \| 7 \| 0-50 \| 29 \| 84 \| 38 \| Nigeria \| NI \| HIV \| \| Ogoina D \| 2020/ Retrospective \| NI \| 51 \| 40 \| 5 \| 0-52 \| 32 \| 31 \| 9 \| Nigeria \| NI \| NI \| \| Noam Erez \| 2019/ Case investigation \| Molecular Method \| 1 \| 1 \| 0 \| 38 \| 38 \| 1 \| 0 \| Israel \| NI \| NI \| \| Matthew R Mauldin \| 2022/ Laboratory study \| Molecular Method \| 5 \| 5 \| 0 \| 32-40 \| 37 \| 4 \| 1 \| UK, Singapore, Israel \| Rodent carcass, Occupational \| NI \| \| Sarah Ee Fang Yong \| 2020/ Case investigation \| Molecular Method \| 1 \| 1 \| 0 \| 38 \| 38 \| 1 \| 0 \| Singapore \| Bushmeat \| NI \| \| Kara N. Durski \| 2018/ Retrospective \| NI \| 3 \| 3 \| 0 \| NI \| NI \| NI \| NI \| Nigeria \| NI \| NI \| \| Agam K. Rao \| 2022/ Case investigation \| Molecular Method \| 1 \| 1 \| 0 \| NI \| NI \| 1 \| 0 \| USA \| NI \| NI \| \| Gemma Hobson \| 2021/ Case investigation \| Molecular Method \| 1 \| 1 \| 0 \| NI \| NI \| 1 \| 0 \| UK \| NI \| NI \| \| Varea Costello \| 2022/ Case investigation \| Molecular Method \| 1 \| 1 \| 0 \| 28 \| 28 \| 1 \| 0 \| USA \| Direct Contact \| NI \| \| Aisling Vaughan \| 2018/ Case investigation \| Molecular Method \| 2 \| 2 \| 0 \| NI \| NI \| 2 \| 0 \| UK \| Consumption of bushmeat \| NI \| \| Lateefat Kikelomo Amao \| 2022/ Laboratory study \| NI \| 25 \| 3 \| 0 \| NI \| NI \| 1 \| 2 \| Nigeria \| Direct Contact \| NI \| \| Dimie Ogoina \| 2019/ Outbreak investigation \| Molecular Method \| 38 \| 18 \| 0 \| NI \| NI \| 17 \| 1 \| Nigeria \| NI \| Syphilis, HIV, Chickenpox \| \| E E Eseigbe \| 2021/ Case investigation \| Molecular Method \| 2 \| 2 \| 0 \| 20 \| 20 \| 2 \| 0 \| Nigeria \| NI \| NI \| \| S O Foster \| 1972/ Laboratory study \| Microscopy \| 1 \| 1 \| 0 \| 4 \| 4 \| 0 \| 1 \| Nigeria \| NI \| NI \| \| Dimie Ogoina \| 2022/ Case investigation \| Molecular Method \| 1 \| 1 \| 1 \| 34 \| 34 \| 1 \| 0 \| Nigeria \| NI \| NI \| \| Womi-Eteng Eteng, \| 2018/ Outbreak investigation \| Molecular Method, Serology. \| 1 \| 1 \| 0 \| NI \| NI \| NI \| NI \| Nigeria \| NI \| NI \| \| Emmanuel Pembi \| 2022/ Case investigation \| Molecular Method \| 1 \| 1 \| 0 \| 30 \| 30 \| 1 \| 0 \| Nigeria \| NI \| NI \| \| Dimie Ogoina \| 2022/ Case investigation \| Molecular Method, Serology \| 1 \| 1 \| 0 \| NI \| NI \| NI \| NI \| Nigeria \| NI \| NI \| \| Barry Atkinson \| 2022/ Case investigation \| Molecular Method, Serology. \| 1 \| 1 \| 0 \| 40 \| 40 \| NI \| NI \| UK \| NI \| NI \| \| Echekwebe \| 2020/ Case investigation \| Molecular method \| 4 \| 4 \| 0 \| 20-32 \| 28 \|  \| 3 \| Benue, Nigeria \| NI \| HIV \| \| Okonkon Ita Ita \| 2019/ Laboratory study \| NI \| 15 \| 6 \| 0 \| 0-59 \| NI \| 8 \| 7 \| Akwa Ibom,  Nigeria \| Bush meat, Rodent, Direct contact, sex \| NI \| \| Solomon Chieloka \| 2019/ Outbreak investigation \| NI \| 8 \| 0 \| 0 \| NI \| 25 \| NI \| NI \| Akwa Ibom, Nigeria \| Bush meat consumption \| NI \| \| Ibegu \| 2020/ Laboratory study \| Molecular method \| 30 \| 11 \| 0 \| 1-43 \| 31 \| 20 \| 10 \| NI \| NI \| NI \|   . |
| Risk of bias in studies | 18 | \| **First author and year of publication** \| **Population under consideration** \| **Sample size adequacy** \| **Subject and setting** \| **Diagnostic tool deployed** \| **Co-author accounted for** \| **Subgroups identified** \| **Quality**  **items met** \| \| --- \| --- \| --- \| --- \| --- \| --- \| --- \| --- \| \| Yinka-Ogunleye, 2019 \| yes \| yes \| yes \| yes \| yes \| yes \| 6 \| \| Ogoina, 2020 \| yes \| yes \| yes \| no \| yes \| yes \| 5 \| \| Erez, 2019 \| yes \| no \| no \| yes \| yes \| no \| 3 \| \| Mauldin, 2022 \| yes \| yes \| yes \| yes \| yes \| yes \| 6 \| \| Yong, 2020 \| yes \| no \| no \| yes \| yes \| no \| 3 \| \| Durski, 2018 \| yes \| yes \| yes \| no \| yes \| no \| 4 \| \| Rao, 2022 \| yes \| no \| no \| yes \| yes \| no \| 3 \| \| Hobson, 2021 \| yes \| no \| no \| yes \| yes \| no \| 3 \| \| Costello, 2022 \| yes \| no \| no \| yes \| yes \| no \| 3 \| \| Vaughan, 2018 \| yes \| no \| no \| yes \| yes \| no \| 3 \| \| Amao, 2022 \| yes \| yes \| yes \| no \| yes \| no \| 4 \| \| Ogoina, 2019 \| yes \| yes \| yes \| yes \| yes \| no \| 5 \| \| Eseigbe, 2021 \| yes \| no \| yes \| yes \| yes \| no \| 4 \| \| Foster, 1972 \| yes \| no \| yes \| yes \| yes \| no \| 4 \| \| Ogoina, 2022 \| yes \| no \| yes \| yes \| yes \| no \| 4 \| \| Eteng, 2018 \| yes \| No \| yes \| yes \| yes \| no \| 4 \| \| Pembi, 2022 \| yes \| No \| yes \| yes \| yes \| no \| 4 \| \| Ogoina, 2022 \| yes \| No \| yes \| yes \| yes \| no \| 4 \| \| Akinson, 2022 \| yes \| No \| no \| yes \| yes \| no \| 3 \| \| Echekwube, 2020 \| Yes \| yes \| yes \| yes \| yes \| yes \| 6 \| \| Ita Ita, 2019 \| yes \| yes \| yes \| no \| yes \| yes \| 5 \| \| Chieloka, 2019 \| yes \| yes \| yes \| no \| yes \| no \| 4 \| \| Ibegu, 2020 \| yes \| yes \| yes \| yes \| yes \| yes \| 6 \| |
| Results of individual studies | 19 | This is only applicable to the studies included in the meta-analysis  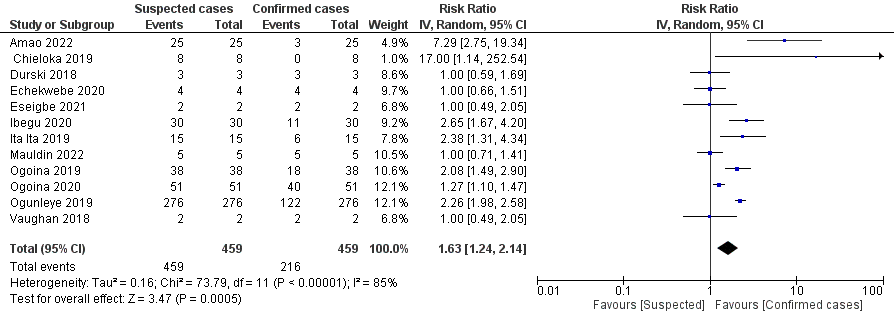 |
| Results of syntheses | 20a | This is already presented in item no 18 |
|  | 20b | 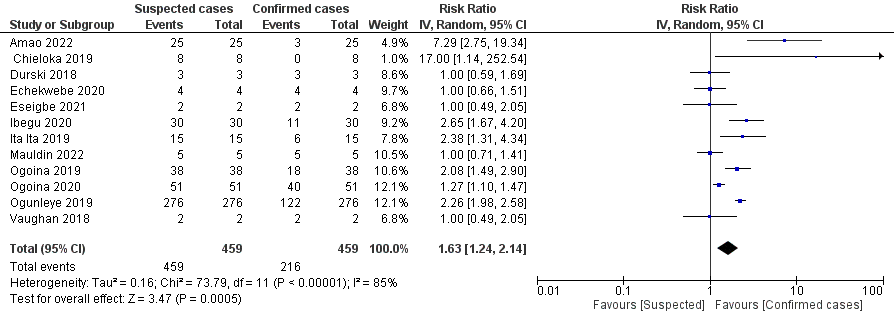  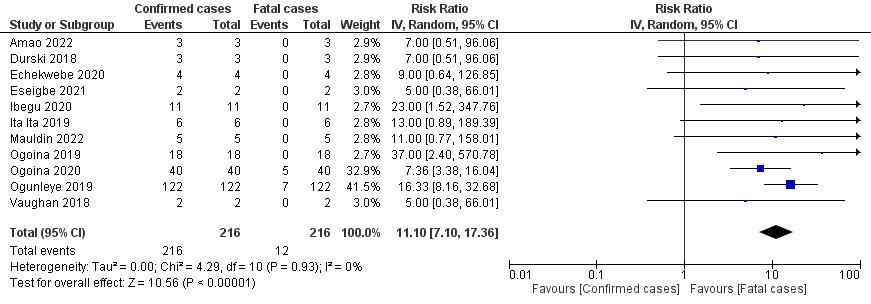  **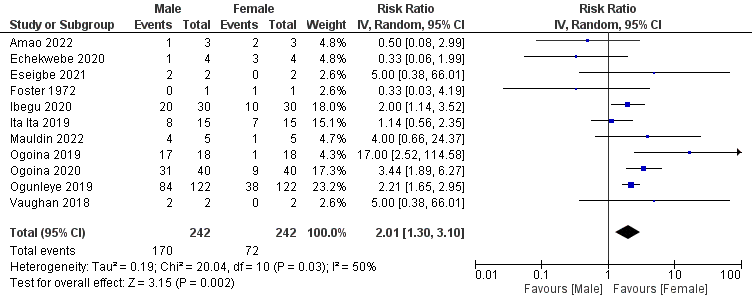** |
|  | 20c | Not applicable |
|  | 20d | Not applicable |
| Reporting biases | 21 | Not applicable |
| Certainty of evidence | 22 | Not applicable |
| **DISCUSSION** | | |
| Discussion | 23a | The findings of this study showed that, over the five years under review, 32 out of 36 states in Nigeria, including the Federal Capital Territory have reported at least a case of Mpox. Most of the cases of Mpox in Nigeria were from the southern part of the country. The results showed progressive spread from southern to the northern regions of the country. Further, positive association were identified between infection and poverty, lack of basic healthcare facilities as well as multiple heterosexual partners. These findings reiterate the need to strengthen and expand on the already existing efforts as well as establish robust multi-sectoral collaboration to better understand the dynamics of Mpox Nigeria. |
|  | 23b | The review could not use many studies because majority of the assessed publications did not meet the inclusion criteria. Also, only 12 out of the 23 included studies could be used for meta-analysis because of missing data. Despite these limitations, our findings have provided valuable insights into the dynamics of Mpox in Nigeria. |
|  | 23c | The review process does not have a major limitation. |
|  | 23d | There is a tremendous gap in the knowledge on the current Mpox outbreak situation in Nigeria. Thus, understanding the dynamics of Mpox will help in mitigating future occurrence of the disease as well as other emerging/re-emerging infectious disease emergencies in Nigeria and other countries with similar socio-cultural and ecological settings, towards averting global spread. Importantly, more needs to be done by the Nigeria Centre for Disease Control (NCDC) in the area of coordination of already existing disease diagnostic infrastructure and getting the states more involved in surveillance systems. Finally, further support and collaboration will be required from international partners and organizations like the World Health Organization, World Organization for Animal Health and the African Centre for Disease Control to strengthen mitigation and preparedness efforts towards averting future outbreaks. |
| **OTHER INFORMATION** | | |
| Registration and protocol | 24a | Not applicable |
|  | 24b | A protocol was not prepared. |
|  | 24c | No amendment to amendments to information was carried out. |
| Support | 25 | No financial support for the study |
| Competing interests | 26 | The authors hereby declare no competing interests. |
| Availability of data, code and other materials | 27 | None of the data presented in this study is publicly available. |
